# Codon-based indices for modeling gene expression and transcript evolution

**DOI:** 10.1016/j.csbj.2021.04.042

**Published:** 2021-04-22

**Authors:** Shir Bahiri-Elitzur, Tamir Tuller

**Affiliations:** a Department of Biomedical Engineering, Tel-Aviv University, Tel Aviv, Israel; b The Sagol School of Neuroscience, Tel-Aviv University, Tel Aviv, Israel

**Keywords:** Gene expression, Transcript evolution, Codon usage bias

## Abstract

Codon usage bias (CUB) refers to the phenomena that synonymous codons are used in different frequencies in most genes and organisms. The general assumption is that codon biases reflect a balance between mutational biases and natural selection. Today we understand that the codon content is related and can affect all gene expression steps. Starting from the 1980s, codon-based indices have been used for answering different questions in all biomedical fields, including systems biology, agriculture, medicine, and biotechnology. In general, codon usage bias indices weigh each codon or a small set of codons to estimate the fitting of a certain coding sequence to a certain phenomenon (e.g., bias in codons, adaptation to the tRNA pool, frequencies of certain codons, transcription elongation speed, etc.) and are usually easy to implement.

Today there are dozens of such indices; thus, this paper aims to review and compare the different codon usage bias indices, their applications, and advantages.

In addition, we perform analysis that demonstrates that most indices tend to correlate even though they aim to capture different aspects. Due to the centrality of codon usage bias on different gene expression steps, it is important to keep developing new indices that can capture additional aspects that are not modeled with the current indices.

## Graphical abstract

**Figure:**
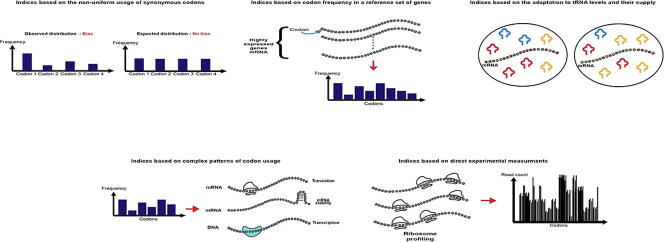

## 1 Introduction and motivation

Synonymous codons are codons that encode for the same amino acid. Despite that, synonymous codons are generally used at different frequencies. This phenomenon can be seen in most genes and organisms, and it is called codon usage bias (CUB).

The composition of codons in the coding region can be affected by mutations, selection [1], and genetic drift [2], [3], [4]. Specifically, the selection can be related to frameshifting and various central intracellular phenomena such as transcription, translation, mRNA stability, RNA and DNA methylation, co-translational folding, splicing, transport, and more (see review in [5], [6], [7]). Thus, techniques for analyzing codon distribution have been used in recent years for many objectives, including studying the evolution of transcripts [8], [9], the biophysics of translation (i.e., the molecular mechanism of translation and its dynamics: e.g., ribosomal movement, tRNA diffusion, etc.) and other gene expression steps [10]. Such techniques also have been used in biotechnology [11], for investigating phenotypic patterns from viruses to bacteria [12], [13], and for studying human diseases [14], [15], [16], [17]*.* A CUB index is a term that describes a measure with a numerical value that aims to have biological meaning (e.g., related to selection or gene expression aspects), and it is based on the codon composition of a gene.

**Fig. 1:**
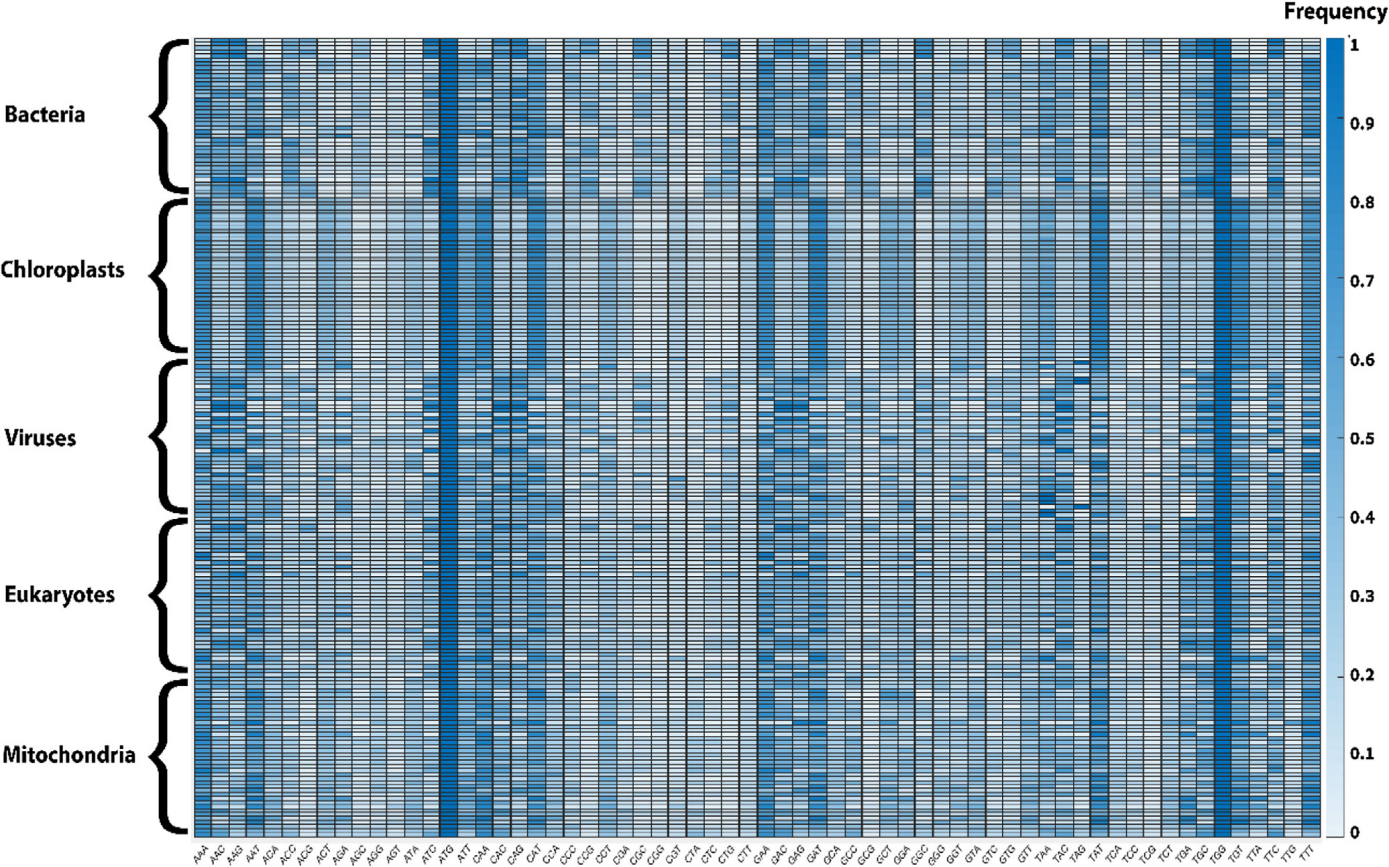
Codon usage of all 64 codons in the different analyzed organisms, organelles, and viruses. The codon usage was calculated using the ratio between the codon's appearances and the relevant synonymous codons’ total appearances. The color bar represents the frequency of each codon (more details in the case study section). As can be seen, there are large differences in codon usage among the analyzed genomes. Equal frequencies can also be seen for codons that code for amino acids with one synonymous codon.

Since CUB can be affected and affect so many phenomena, as mentioned previously, codons frequencies vary significantly among different organisms, organelles, and viruses. We examined codon frequencies in different organisms, organelles, and viruses chosen uniformly from the tree of life and from different viral groups (see Fig. 1).

It is easy to see different codon distribution patterns in the examined groups; this may be related both to changes in the mutational bias and to the differences in the fitness effect of codons. The high variation in bacteria, viruses, mitochondria, and eukaryotes is related to the diverse genomes we analyzed, including bacteria from different phyla, different types of viruses, and various eukaryotes and mitochondria. Low variability can be seen in the chloroplasts genomes. The chloroplasts are more conserved relative to other types of genomes that are presented since chloroplasts are evolutionary closer; in addition, they have the same role and function (i.e., conducting photosynthesis), their genome is relatively small, and it is known that the chloroplast genome has a highly conservative nature and slow evolutionary rate [18].

CUB indices have many significant advantages:

First, the estimations of codon usage by different indices are usually very easy and computationally efficient. Many indices are based only on the analyzed organism's genome (without the need for performing additional experiments); thus, they can be implemented on any organism with a sequenced genome (unicellular and multicellular organisms [19], [20]). Finally, as depicted in the following sub-sections, many times, these indices estimate aspects that cannot be experimentally measured with any technique that exists today.

Today there are dozens of different CUB indices. This review aims to summarize the different types of indices that exist today, explain their advantages, objectives, and compare them.

**Fig. 2:**
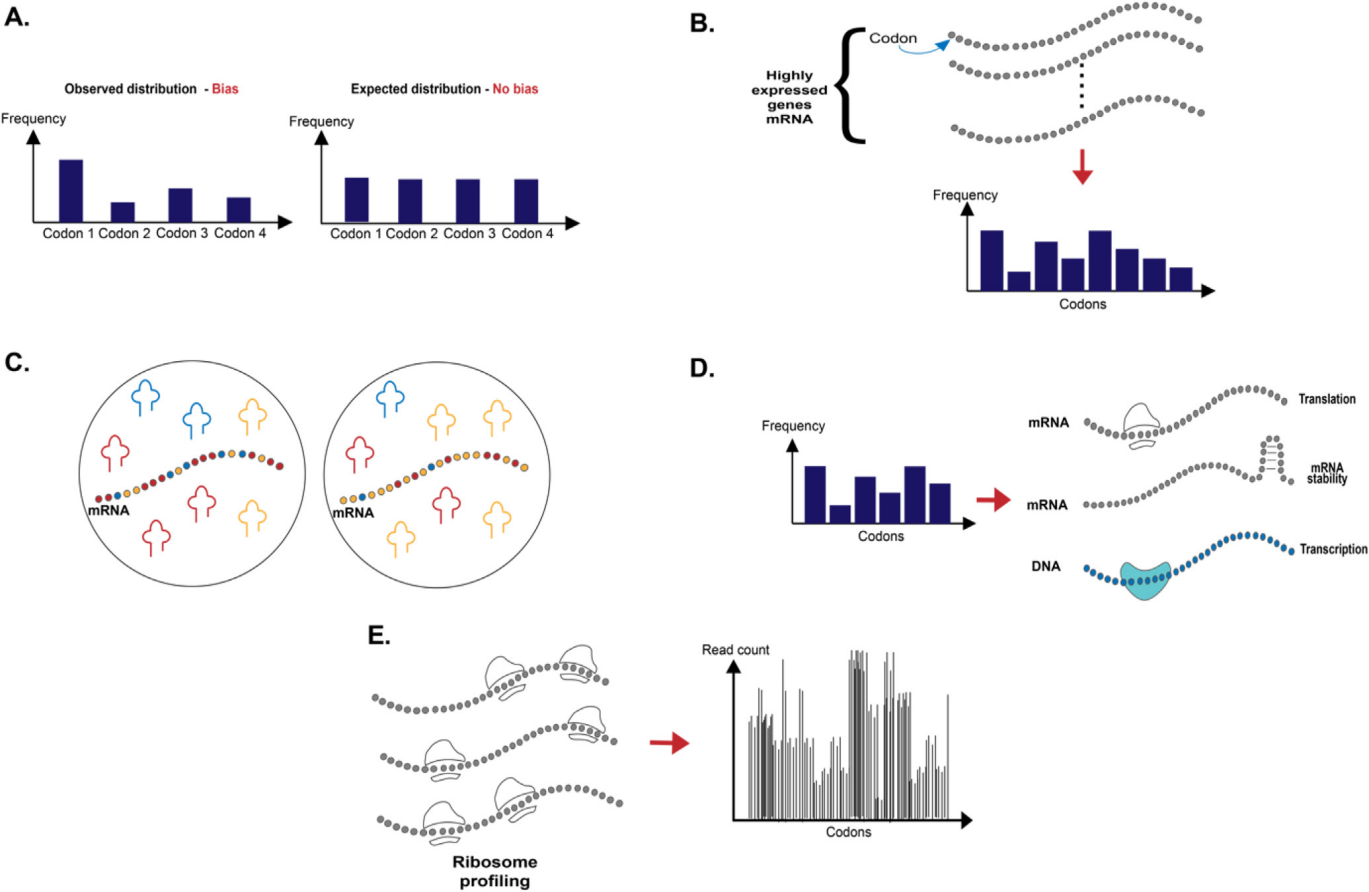
*Different types of CUB* indices *examined in the paper****. A.****Indices that are based on the non-uniform usage of synonymous codon.****B.****Indices based on codon frequency in a reference set of genes. To deal with alternative splicing when using such indices, the longest isoform of the gene is usually considered.****C.****Indices that are based on the adaptation to the tRNA levels, and their supply.****D.****Indices that consider complex patterns of codons that affect translation, transcription, and mRNA stability.****E.****Indices that are based on a direct experimental procedure such as ribosome profiling.*

There are a few types of CUB indices which are discussed here (see Fig. 2): this includes “simple” indices based only on the non-uniform usage of synonymous codon, indices that try to capture biophysical aspects of gene expression such as tRNA adaptation, indices that are based on experimental procedures, and even indices that aim to capture patterns that are more complex than a single codon distribution. The indices discussed in this paper are important as they correlate and relate to how various gene expression stages are encoded in the coding region. In addition, they consider various factors in the cell that can influence transcripts evolution (i.e., tRNA, highly expressed genes, and more). Moreover, some of these indices enable exploring novel aspects and regulatory signals encoded in the coding region that may be longer than one codon.

There are review papers that aim to understand the CUB mechanism, affect, and causes [21], [22], [23]; However, there are not many previous review papers regarding CUB indices. These earlier reviews are helpful, but they summarize a limited number of indices (usually 5–7) [24], [25], [26] without considering all possible or at least indices from different types.

Our review briefly summarizes and discusses known and less known CUB indices, aiming to provide a useful basic guideline for researchers in the field. We specifically focused on different approaches to measure CUB and discuss each approach's advantages and disadvantages.

## 2 Indices that are based on the non-uniform usage of synonymous codon

This group of indices computes the deviation of codon usage frequency from “uniform” distribution or expected “background” distribution (see Fig. 2A). An increased CUB in a certain gene or genomic region may suggest that it undergoes selection for various features that affect its expression levels (including translation, transcription, mRNA stability, co-translational protein folding, and more). These indices are usually monotonic with the uniformity level of the codon usage or codon frequency. The two extremes are cases where 1) only one codon is used and 2) all codons are used in the same frequency.

Different indices use various normalization for getting the exact value: for example, relative synonymous codon usage (RSCU) [1] compares the codon frequency to the one expected under uniform distribution while considering both the different number of synonymous codons for various amino acids and the differences in amino acid frequency. The Effective number of codons (ENC) [27] is computed similarly to the computation of effective population size in population genetics, aiming to provide the extent of codon preference in a gene while considering amino acid degeneracy levels. In a basic manner, the ENC estimates the total number of different codons used in a sequence. The Intrinsic codon bias index (ICDI) [28] is inspired by both the RSCU and the ENC. It gives a value between 0 (equal usage of all codons, i.e., no bias) and 1 (one codon per amino acid, i.e., extremely high bias) to each gene. According to Arif Uddin [25], a value greater than 0.5 indicates high bias, whereas a value lowers than 0.3 means little bias. Shields et al. suggested an index which is based on the chi-squared (*χ*^2^) test, and it is called the ‘scaled’ *χ*^2^ [29]; the *χ*^2^ is defined as the deviation from equal usage of a codon within the synonymous group normalized by the total appearances of the codon (excluding AA with one codon).

An additional index of deviation from a uniform distribution is based on the Shannon entropy; the Synonymous codon usage order (SCUO) [30], [31], [32] is based on the normalized difference between the maximum entropy and the observed entropy, that after some normalizations yield a measure between 0 (maximum bias) to 1 (no bias).

See more indices in Table 1.

**Table 1:** Codon bias Indices that are based on measures of the non-uniform usage of synonymous codon.

Index type: The non-uniform usage of synonymous codon						
Index name	Specific characteristics	Uses	Values	Analyzed organism	Online tool	Reference
ENC (effective number of codons)	Similar to the computation of effective population size in population genetic. Considers amino acid degeneracy level and calculates the total number of different codons used in a sequence.	Investigate codon usage patterns across genes and in different organisms. ENC plots provide a visual display of CUB variation for a set of genes. It can easily be adapted to study genes that do not use the 'universal' genetic code.	Range: 20–61. A value of 20 indicates that only one codon is used for each AA, while a value of 61 is when all synonymous codons are used with equal probability.	*Human*, *Scerevisiae*	[73 ]	[27]
RSCU (relative synonymous codon use)	Based on the ratio between the observed number of codons and the number of times the codon would be observed if the synonymous codon usage was completely random.	Acts as a codon weight for many indices requiring the codon count to be normalized into codon frequency and to remove the dependence on gene length. RSCU can be used to find optimal codons and understand evolutionary processes.	For average synonymous codon usage, the RSCU is 1. For codon usage more infrequent than the average codon usage, the RSCU is less than one, and for more frequent usage than the average for the amino acid, the RSCU is greater than 1.	*E.coli*	[73 ]	[1]
CPB (codon preference bias)	Based on the multinomial and Poisson distributions. It can be applied to a relatively short piece of sequence. Not used often, the method is quite theoretical.	Scan DNA sequences and measure the strength of codon preference. It can be used to detect bona fide coding sequences.	A higher value indicates more bias towards optimal codons.	*E.coli*		[74]
The ‘scaled’ *χ*[2]	Calculates the deviation from equal usage of a codon within the synonymous group divided by the total number of codons in the gene using the chi-squared (*χ*^2^) test.	Measures the bias in silent DNA divergence codon usage.	Ranges from 0 to 1. Higher value indicates a stronger bias.	*D.melanogaster*	[75 ]	[29]
P (codon preference)	Measures the likelihood of a particular set of codons to a predetermined preferred usage. P is computed for all three reading frames.	It is useful for locating genes in sequenced DNA, predicting the relative level of their expression and for detecting DNA sequencing errors resulting in the insertion or deletion of bases within a coding sequence.	A higher value indicates a more frequent use of preferred codons.	E.coli, *E.amylovora*, *S.typhimurium* and *S.cerevisiae*	[76 ]	[77]
RCBS (relative codon bias strength)	Measures the difference of the observed frequency of a codon from the expected frequency under the hypothesis that the frequency of codons is only affected by the frequencies of single nucleotides.	Predicting gene expression levels from RSCU. Useful for comparing different sets of gene.	A value close to 0 indicates a lack of bias for the codons.	*E.coli*		[78]
ICDI (intrinsic codon deviation index)	Uses the RSCU and the degeneracy of amino acid in the sequence. It gives equal weight to all amino acids included.	Estimate codon bias of genes from species in which optimal codons are not known. It can help predict gene functionality.	Values between 0 (equal usage of all codons) and 1 (one codon per amino acid)	*S.cerevisiae*	[79 ]	[28]
Dmean (mean dissimilarity-based index)	Quantifies the level of diversity in synonymous codon usage among all genes (or a subset of genes) within a genome. The index is based on the average Pearson correlation between all pairs of genes normalized vectors of codon frequencies.	It is used to measure the diversity level of codon usage among genes. This index can be applied to other compositional features such as amino acid usage and dinucleotide relative abundance as a genomic signature. This index can improve the understanding of compositional diversity among genes.	Lower average correlation values, indicate low diversity level in the use of codons.	268 bacterial genomes such as *B.subtilis*	–	[80]
Ew (weighted sum of relative entropy)	Measures the degree of deviation away from equal codon usage using a weighted relative entropy of each amino acid. It is defined as the sum of each amino acid's relative entropy weighted by its relative frequency in the sequence.	Allows to avoid some amino acid usage biases and to obtain quantitative information about the degree of overall synonymous codon usage bias of a gene.	Ranges from 0 (maximum bias) to 1 (no bias).	*A.aeolicus, B.subtilis, B.burgdorferi*, *E.coli*, *H.influenzae*, *H.pylori*, *M.tuberculosis*, *M.genitalium*, *P.hori-koshii*	–	[81]
SCUO (Synonymous codon usage order)	Measures the deviation from uniform distribution based on the Shannon entropy. It uses the normalized difference between the maximumentropy and the observed entropy.	This index compares the synonymous codon usage across different organisms.	Ranges from 0 (maximum bias) to 1 (no bias).	*E.coli*	[31 ]	[30], [31], [32]
DCBS (directional codon bias score)	A correction to the RCBS that considers over and underrepresented codons.	It can be used to predict gene expression levels from Relative Codon Usage Bias. It is useful for comparing different sets of gene.	A value close to 0 indicates a lack of bias for the codons.	*E.coli*, *S.cerevisiae*	–	[41]
MILC (Measure independent of length and composition)	Measures the different usage of codons based on a log-likelihood ratio of the expected and observed number of codons.	It is used for the prediction of expression levels by taking the ratio of the MILC of a gene to the MILC of a reference set of highly expressed proteins.	A higher value indicates stronger bias.	*E. coli,*, *S.cerevisiae*, *B.subtilis*, *Synechocystis*, *P.falciparum*	-	[82]
MCB (Maximum-likelihood codon bias)	Estimates that bias in codon usage using the weight of each amino acid which is estimated by the likelihood of occurrence of each amino acid given its frequency and codon degeneracy.	Designed to account for background nucleotide composition and can also be adapted to correct for di-nucleotide biases. It can be used to estimate ancestral codon usage bias and genetic population analysis.	A higher value indicates a stronger bias.	*Human*	–	[83]
CDC (Codon Deviation Coefficient)	Based on the cosine distance metric between the expected and the observed codon usage. The authors suggest estimating the significance of this index by using bootstrapping.	It is useful for determining comparative magnitudes and patterns of CUB for genes or genomes with diverse sequence compositions.	Ranges from 0 (no bias) to 1 (maximum bias)	Unicellular and multicellular genomes.	–	[84]
MRI (﻿Mutational Response Index)	Based on the difference between the scaled chi-square test and its corrected form.	Measures the extent of codon bias attributable to mutational pressure.	﻿Positive MRI values indicate a response, whereas negative values for MRI imply a codon usage contrary to directional mutation pressure.	*R.norvegicus*,*M.musculus*, *Human*	–	[85]

These indices' advantages include the fact that they are very simple to implement and are based only on coding sequences. They specifically do not require any prior knowledge of gene expression' biophysics and molecular mechanisms and do not need any additional measurements.

Their disadvantages include the fact that they are not sensitive to patterns different from a single codon distribution. They may miss specific biophysical aspects of gene expression, such as the contribution of different gene expression steps and factors to the observed bias, and they are not condition-specific.

Therefore, it is useful to use this type of indices when only the organism's genome is available.

In this type of indices, the most used indices according to our review based on the usage and number of citations, are ENC and RSCU.

## 3 Indices based on codon frequency in a reference set of genes

The indices in this group are based on the comparison to codon frequency in a reference set of genes (see Fig. 2B). The reference set of genes can be, for example, highly expressed genes (and thus assumed to undergo stronger selection to include more optimal codons), ribosomal genes (also known to be highly expressed), or genes that are expressed in a specific condition or tissue. In all of these indices, coding sequences that include codons that are more similar to the codons in the reference set will get a higher score. The differences among the indices are related to the way that the similarity score is computed.

For example, in the Codon adaptation index (CAI) [33], a weight is calculated for each codon, based on its RSCU among synonymous codons in the reference set (1/0 are the maximal/minimal weights of a codon, respectively). These weights are then used to compute a score for a coding region by using the geometric mean of the codons weights composing the coding region. The Frequency of optimal codons (FOP) [34], [35] is a lower resolution version of the CAI where the score of a gene is calculated by the ratio of the number of optimal codons (the most frequent synonymous codon) to the total number of codons. The score range is between 1 (usage of only the most frequent synonymous codons in the reference set) and 0 (non-usage of the most frequent codons from the reference set). The Codon bias index (CBI) [36] measures the extent that preferred codons are used in a gene. It is calculated similarly to FOP but uses the expected usage as a scaling and normalization factor such that the score is between 1 (all codons are preferred) and −1 (non-codon is preferred) while the FOP is between 0 and 1.

The codon enrichment correlation (CEC) [37] measures the fit of a specific ORF's codon frequency in a reference set of ORFs using the following steps. First, each codon's frequency in the target ORF is normalized by its expected distribution when assuming uniform frequency usage of synonymous codons*.* For the reference set codons, each codon's frequency is the relative frequency in all ORFs in the reference set. Next, the correlation between these two vectors of normalized codon frequency is computed.

An additional index, called GCB [38], tries to deal with the need of defining a reference set (usually of highly expressed genes as in the case of CAI) via iteratively choosing genes with the highest (temporary) GCB score as the reference set till convergence. Each codon weight is defined as the ratio between the codon frequency in the reference set and the codon's mean frequency in the entire genome. These weights are then used for computing a score for a coding region by using the geometric mean of the codons weights composing the coding region. Based on this score, a new (temporary) reference set with the highest score is defined as an input of the next iteration.

Tissue-Specific Codon and Codon-Pair Usage Tables (TissueCoCoPUTs) are comprehensive computational resources for tissue-specific codon, codon-pair, and dinucleotide frequency usage. The mentioned frequencies are calculated in the simplest manner. For example, to calculate the frequency of a codon in a tissue, the codon's total appearances in the tissue transcripts are divided by the total number of codons in the tissue transcripts. When using this index, it is possible to determine translation kinetics and efficiency across different tissues and predicting the propensity of synonymous mutations to cause disease [39].

See more indices in Table 2.

**Table 2:** Codon bias indices based on codon frequency in a reference set of genes summary.

Index type: Codon frequency in a reference set of genes						
Index name	Specific characteristics	Uses	Values	Analyzed organism	Online tool	Reference
CAI (Codon adaptation index)	Assess the relative merits of each codon. A gene score is calculated from the frequency of use of all codons in that gene based on a reference set of genes. The index assesses the extent to which selection has been effective in molding the pattern of codon usage.	Predict protein expression levels.	A higher score for genes that tend to includes codons that are more frequent in highly expressed genes (range from 0 to 1).	*S.cerevisiae*	[73], [86], [87]	[33]
FOP (Frequency of optimal codons)	The frequency of optimal codons of a gene is the ratio of the number of optimal codons to the total number of codons. The optimal codons can be defined according to nucleotide chemistry, codon usage bias, or tRNA availability.	Predict protein production levels and shows the optimization level of synonymous codon choice.	The FOP values range from 0 (optimal codon never appear) to 1 (optimal codon always appear).	*S.cerevisiae*, *E.coli*, *C.* *species*	[73 ]	[34], [35]
ΔFop (difference in frequenciesof optimal codons between constrained and nonconstrained codons)	Measures the difference in frequencies of optimal codons used in a gene at codons related to AA that are evolutionary conserved vs. codons related to AA that are not evolutionary conserved. It is based on the assumption that higher codon bias exists in sites related to conserved AA.	It is a useful index to test directly the hypothesis of selection for translational accuracy and selection for the fidelity of protein synthesis.	A significant positive value indicates that optimal codons use is higher at constrained codons than at nonconstrained ones.	*C.elegans*, *Human*	–	[88]
CBI (Codon bias index)	Measures the usage of optimal codons using the ratio between the number of optimal codons in a gene and the total number of codons in a gene. It uses the expected usage as a scaling factor.	Reflects the presence of components with high CUB in a particular gene. It can describe foreign gene expression in a host.	Values range between −1 and 1. A value of 1 means only preferred codons are used, zero means random choice and less than zero implies greater use of nonpreferred codons.	*S.cerevisiae*, *M.tuberculosis*	[73]	[36]
B (Codon usage bias)	Based on the frequency weighted sum of distances of the relative codon usage frequencies between two sets of genes.	It is used to infer the expression level by comparing the fraction of the distance of the query set with respect to all genes over the distance to a reference set, or a linear combination of reference sets.	The possible values range from 0 to 2, rarely exceeding 0.5. Higher value indicates codon usage differences between the two sets of genes.	*Human*, *E.coli*	–	[89], [90]
CEC (Codon-enrichment correlation)	The linear correlation coefficient of the codon enrichment between the ORF and a reference set of genes. The enrichment of each codon is defined as the ratio of its frequency among the named ORFs by its expected frequency in random sequences.	Together with expression data, CEC can be used to identify spurious open reading frames and can be used to detect incorrectly assigned ORFs that are not coding for a protein.	If a sequence is not detected experimentally and the CEC is lower than the cutoff value, then the ORF is designated as spurious.	*S.cerevisiae*	–	[37]
rCAI (relative codon adaptation index)	This index is similar to CAI, but defines each codon weight by normalizing it with the codon usage in the + 1 and + 2 reading frames.	It is used to discriminate between highly biased and unbiased genomic regions. It can detect codon bias patterns in overlapping genes and capture local codon bias patterns.	rCAI ranges from 0 to an upper limit, which corresponds to an imaginary gene consisting only the maximum-weighted codons.	*E.coli*, *L.lactis*, additional bacteria [91]	–	[92]
GCB	Defines iteratively the reference set for weighting the codons frequency.	It can be used to identify hypothetical genes.	A higher GCB value is assigned to genes that tend to include codons that are more frequent in the reference set.	*S.cerevisiae*, *E.coli*, additional bacteria	-	[38]
ITE (index of translation elongation)	It is similar to CAI, but for each codon, a weight is calculated based on its frequency among NNR and NNY codons subfamilies in the reference set. The reason for separating synonymous codons into R- and Y-ending codon subfamilies is that different tRNAs typically translate them and they are subjected to different mutation bias.	It can predict protein expression levels.	A higher score for genes that tend to includes codons that are more frequent in highly expressed genes (range from 0 to 1).	Unicellular and multicellular organisms	-	[93]
TissueCoCoPUT (Tissue specific Codon and Codon-Pair Usage Tables)	Measures the observed/expected codon, codon pair, and dinucleotides frequency and calculates the difference in different tissues.	It can be used for determining translation kinetics and efficiency across tissues.	Higher values indicate a higher compatibility to a certain tissue.	*Human*	[39 ]	[39]
RCA (Relative codon adaptation)	This index uses a given reference set to compute observed and expected codon frequency. It considers the underlying nucleotide distribution at the third position in the codon.	It can predict protein expression levels.	A higher score for genes that tend to include codons that are more frequent in highly expressed genes.	*S.cerevisiae*,*C.elegans,D.melanogaster*,*E.coli*	–	[94]
COUSIN (Codon usage similarity index)	This index compares the CUB of a query against those of a reference and normalizes the output over a Null Hypothesis of random codon usage.	It can be used to identify differential heterogeneity between and within genomic data sets.	A COUSIN score of 1 indicates that the CUB in the query is similar to the reference data set; A COUSIN score of 0 indicates that the CUB in the query is similar to the Null Hypothesis (i.e., equal usage of synonymous codons).	*E.coli*, *S.coelicolor*, *A.thaliana*, *S.cerevisiae,P. falciparum*, *G.gallus*, *Human*, *M.musculus*	[75 ]	[75]
SCCI (Self Consistent Codon Index)	This index is based on the CAI, but the chosen reference set is the most biased set of genes.	It can be used to predict gene expression levels, to guide regulatory circuit reconstruction, and to compare species.	A higher score for genes that tend to includes codons that are more frequent in the reference set of genes (range from 0 to 1).	*M.pulmonis*, *M.tuberculosis*, *T.pallidum*, *H.pylori*, *P.aeruginosa*, *B.burgdorferi*, *H.influenzae*, *S.enterica*, *S.aureus*, *L.lactis*, *B.subtilis*, *E.coli*, *S.cerevisiae*, *C.elegans*, *D.melanogaster*	–	[95], [96]

This type of indices' main advantages are that they are very simple to compute (usually involves calculating the frequency of a codon appearance in sets of genes and geometric mean). In many cases, these indices can be based only on the organism's genome without additional knowledge or measurements. Also, the prior knowledge that is needed is minimal (only a reference set).

The disadvantages include the fact that the indices are based on simple patterns of single codons and cannot capture complex patterns (e.g., based on more than single codons distribution) and cannot estimate the direct effect of specific biophysical aspects (e.g., different gene expression steps). In addition, the reference set needs to be chosen, and the results can vary when using different reference sets.

Therefore, it is useful to use this type of indices when there is a known reference set, and the organism genome is available.

In this type of indices, the most used indices according to our review based on the usage and number of citations, are CAI and FOP.

## 4 Indices based on the adaptation to tRNA levels and their supply

tRNA molecules are major factors that are believed to affect translation elongation at the genomic level. Thus, many indices are based on the adaptation of codons to the cell's tRNA levels (see Fig. 2C). Previous studies have suggested that intracellular tRNA levels correlate with codon usage and amino acid composition in many prokaryotic and eukaryotic species [22], [40]. This correlation optimizes the efficient use of resources in the cell.

While there are studies that suggest that in some organisms (e.g., multicellular organisms), there is a low correlation between CUB and tRNA levels [21], others have demonstrated that such a correlation appears in most organisms, including multicellular [22], [40], [41], [42]. Nevertheless, as the indices described in this section check how much the codons of a gene(s) fits the tRNA pool, they can be implemented and yield biological conclusions on any gene in any organism even if there is no strong global correlation between CUB and tRNA levels in that organism. Beyond this relation between codon composition and translation speed lies a more complex set of phenomena that link codon usage and tRNA abundance to various aspects of translation regulation. Translation regulation is affected by tRNA modifications, codons order, codons usage with rare cognate tRNAs, rRNA-mRNA interactions, wobble interactions, mRNA folding, and more [40], [43], [44], [45], [46], [47], [48]. Moreover, previous results suggest that codon usage and tRNA abundance coevolve [49]: changes in tRNA levels trigger changes in codon usage and vice versa. Therefore we also report indices that generally aim to capture in a simple manner the efficiency of translation elongation based on tRNA-centric view. Among others, different indices in this group consider the amount of each tRNA in the cell, the 'competition' of codons on tRNAs, the wobble/no-wobble interaction efficiency of tRNAs and codons, and more.

For example, the tRNA adaptation index (tAI) [50] computes a weight (between 0 and 1) for each codon based on the number of tRNAs available in the cell that recognize the codon (more tRNAs will result in a higher weight) and the efficiency of the interaction between the different tRNAs and different codons (the efficiency of interaction was calculated by maximizing the correlation between mRNA levels and tAI values in *S. cerevisiae*). The score of a coding region is the geometric mean of the weights of all its codons. The species-specific tAI (stAI) [41] is based on the tAI. However, in this case, the interaction efficiency between a tRNA and a codon is fitted to each organism separately using optimization algorithms that optimize the correlation between tAI and an CUB index.

Some indices consider not just the tRNA amount (supply) but also the abundance of the relevant codons (demand). The normalized translation efficiency (nTE) [51] is an index that considers both the supply and the demand by dividing each codon weight (based on the tAI after some normalizations) and the relative frequency of the relevant codon in all mRNAs. Another example of an index that measures the adaptation of codons to tRNA levels is the P1 index [52]. The P1 index considers the effect of tRNA availability and “tRNA competition” on elongation via estimating the average number of tRNA-codon interactions necessary for correct recognition of a step in the elongation cycle. Since rare tRNAs will approach the ribosomal A site with lower probability (assumed to be related to their frequency), the expected time to recognize their corresponding codon is higher.

See more indices in Table 3.

**Table 3:** Codon bias indices based on adaptation to the tRNA levels and their supply summary.

Index type: Adaptation to the tRNA levels and their supply						
Index name	Specific characteristics	Uses	Values	Analyzed organism	Online tool	Reference
tAI (tRNA adaptation index)	Computes a weight for each codon based on the tRNA copy number and the properties of anticodon–codon interaction.	It can be used to measure translation efficiency.	Higher values for genes that tend to includes codons that are more adapted to the tRNA pool (range from 0 to 1).	*H.pylori*, *S.cerevisiae*, *Human*, *A.thaliana*, *M.musculus*, *P.falciparum*, *N.crassa*, *D.melanogaster*, *C.elegans*	–	[50]
stAI (species-specific tRNA adaptation index)	Species-specific tAI. This index estimates the tAI codon – anti codon interaction weights without the need for gene expression measurements.	It can be used to measure translation efficiency .	Higher values for genes that tend to includes codons that are more adapted to the tRNA pool (range from 0 to 1)	Unicellular and multicellular organisms.	[97 ]	[41]
P1 index	Measuresthe average number of tRNA–codon interactions necessary for a correct recognition for a step for a correct recognition for a stepin the elongation cycle. It is based on protein synthesis dynamics.	It can measure the influence of tRNA availability on the gene translation.	Lower values are related to genes that are optimized for a small number of tRNA discriminations and are often highly expressed.	*E.coli*	–	[52]
P2 index	This index is based on the fraction of pyrimidine-ending codons that have intermediate strength.	It can measure the bias for anticodon–codon interactions of intermediary strength.	A high value for highly expressed genes and low for lowly expressed genes.	*E.coli*	–	[52]
nTE (normalized translational efficiency)	This index considers both the supply and the demand of a codon by computing for each codon a weight which is based on the tAI and the relative frequency of the relevant codon in all the mRNAs.	It can measure translation efficiency.	Codons are considered optimal if the relative availability of cognate tRNAs exceeds their relative usage resulting in a higher value.	*S.cerevisiae*, *C.glabrata,* *D.hansenii*, *K.lactis*, *S.bayanus*, *S.kluyveri*, *S.mikatae*, *S,paradoxus*, *S.pombe,* *Y.lipolytica*	–	[51]
compAI (Competition Adaptation Index)	Based on the competition between cognate and near-cognate tRNAs during translation. It is defined as the harmonic average of the relative adaptiveness of the gene codons.	It can provide information about the speed of protein synthesis.	Values between 0 and 1, where values close to 0 indicate higher competition, and therefore a low translation rate.	*E.coli*	–	[98]
TPI (tRNA-pairing index)	A Measure of synonymous codon ordering comparing of the number of changes of tRNA in a coding sequence to the total number of expected changes given a random distribution of the existing codons.	It can be used to measure tRNA reusage of a gene.	A value of 1 means a completely ordered sequence of the codons by their decoding tRNA. A value of −1 means a completely unordered sequence.	*S.cerevisiae*	–	[99]

The main advantages of this type of indices are that they are computationally efficient and based on the biophysical aspects (at least partially) of the translation process (considering factors in the cell that can influence translation, such as tRNA levels).

Their disadvantages include the fact that they are not based only on the genome but also require additional information such as estimations of tRNA levels, codon-tRNA interactions efficiency, and mRNA levels. They are also based on single codon resolution and do not capture complex patterns and other gene expression aspects except translation elongation. Since tRNA levels are often not available, usually tRNA copy numbers are used as a proxy for tRNA abundance in the cell [34], [50], [53], [54], [55]. This approximation is problematic in more complex organisms that demonstrate tissue-specific differences in the expression of tRNA genes [56]. In addition, organisms with larger genomes tend to have higher tRNA genes redundancy, which would decrease selection for specific codons; this may affect our capability to successfully use such indices for more complex organisms [50].

Therefore, it is useful to use this type of indices when additional information related to tRNA levels is available and when the study's focus is the effect of tRNAs on elongation.

In this type of indices, the most used index according to our review based on the usage and number of citations, is tAI.

## 5 Indices based on 'complex' patterns of codon usage

In recent years, we have learned that sequences longer than one codon in the coding region may include regulatory codes that are related to various aspects of gene expression and intracellular processes (and not only translation); thus, indices that can capture such aspects, which can't be computed solely based on single codon distributions, are needed (see Fig. 2D). These indices capture complex signals that are partially affected and affect codons. A change in the codon composition can affect the such long patens and these pattern induces a selection pressure on codon composition. We thus believe that the notion of codon-based index should also include measures with higher statistics than only single codons.

For example, under the assumption that evolution shapes coding sequences to improve gene expression regulation, if a gene shares longer sequence blocks with the rest of the genome, it is possibly more optimized and more highly expressed. The ChimeraARS [57] index (average repetitive substring) is based on the coding region's tendency to include long substrings that appear in other coding sequences and assume to be regulatory regions. If the coding region has more such longer common substrings (which are usually longer than three nucleotides, i.e., a codon) with other coding sequences, the ChimeraARS score is higher. It is known that some regulatory signals are expected to appear in specific regions of the coding region (e.g., at the beginning or the end). The ChimeraUGEM [58] index aims at capturing this aspect by considering both the size of the commonly detected substring and its location within host genes. The input to these two indices can be based on an alphabet of codons (and in this case, the substrings have lengths in multiples of 3) or nucleotides (in this case, the lengths are not in multiples of 3). We believe that alphabet of nucleotites is also relevant since these patterns are partially encoded by codon usage.

Another example of an index that captures a signal longer than a single codon is the effective number of codon-pairs (ENcp) [59]. It is defined analogously to ENC, with an addition of a square root to the equation and measures the bias of codon pairs distribution. Additional codon pairs bias index is the codon pair score (CPS) [60]. The CPS is defined as the observed ratio's natural logarithm over the expected number of occurrences of a particular codon pair in all protein-coding sequences of a species. A negative CPS value means that a specific codon pair is underrepresented, whereas a positive CPS value indicates that a particular codon pair is overrepresented. Codon pairs that are equally under or overrepresented have a CPS equidistant from 0. From the CPS, a gene score of codon pair bias can be calculated by the average of the CPSs of all codon pairs in the gene [61].

Another type of complex codon usage pattern is estimated by the index 'synonymous codon usage bias maximum likelihood estimation' (SCUMBLE) [62]**.** This index aims to model various sources of codon usage bias in a gene and it is based on a probabilistic model inspired by statistical physics. The maximum likelihood approach is used for estimating the model parameters. SCUMBLE separates the codon usage variation into distinct sources of bias and captures each gene's average codon usage.

See more indices in Table 4.

**Table 4:** Codon bias indices based on complex patterns of codons summary.

Index type: Complex patterns of codon usage						
Index name	Specific characteristics	Uses	Values	Analyzed organism	Online tool	Reference
ChimeraARS	Based on the tendency of a coding region to include long substrings that appear in other coding sequences and assumed to be regulatory regions.	It is used to predict gene expression and estimate the adaptability of an ORF to the intracellular gene expression machinery of a genome (host).	A higher score is related to higher adaptivity of the gene to the host genome.	*E.coli*	[58 ]	[57]
ChimeraUGEM	Region and position specific ChimeraARS.	It is used to predict gene expression and estimate the adaptability of an ORF to the intracellular gene expression machinery of a genome (host).	A higher score is related to higher adaptivity of the gene to the host genome.	*E.coli*	[58 ]	[58]
GC3 (GC content at the third position of synonymous codons)	Measures the GC content at the third position of synonymous codons. It tries to capture the fact that optimal codons in highly abundant proteins tend to have pyrimidines at the third position.	It can predict protein expression levels.	Values range from 0 to 1, a higher value correlates with highly expressed genes.	*C.elegans*	[73 ]	[100]
SCUMBLE (Synonymous codon usage bias maximum-likelihood estimation)	The model parameters are estimated by the maximum likelihood approach. This index captures nonlinearities betweenexpression levels and codon usage.	It can identify different sources of bias in various genomes and estimate the other sources' degree of contribution and their effects on a gene..	A higher value for a source indicate a stronger bias relates to it.	*S.cerevisiae*, 325 prokaryotes genomes such as *B.subtilis* and *E.coli*	–	[62]
SEMPPR (Stochastic evolutionary model of protein production rate)	Based on the stochastic evolutionary model, which assumes that selection to reduce the cost of nonsense errors drives the evolution of codon bias. This index generates a posterior probability distribution for the protein production rate of a given gene.	It can be used to predict protein production rate and expression levels.	Genes with low production rates will have a smaller difference in the energetic usage between the highest peak and lowest probability distribution valley than those with high production rates.	*S.cerevisiae*	–	[101]
Codon volatility	Measures the proportion of the point-mutation neighbors of a codon that encodes different amino acids. It is based on the observation that codons differ with respect to the likelihood that a point mutation will cause a nonsynonymous mutation.	It can be used to estimate selective pressures. It can be used to scan an entire genome to find genes that show significantly more, or less, pressure for amino-acid substitutions than the genome as a whole.	A gene that contains many residues under pressure for amino-acid replacements will exhibit on average elevated volatility.	*M.tuberculosis*, *P.falciparum*	–	[102]
ENcp (effective number of codon-pairs)	Measures codon pairs bias. It is defined analogously to ENC.	Can be used to Investigate codon-pairs usage.	Ranges from 20 (very biased) to 61 (not biased at all).	1,275,531 individual species	[39 ]	[59]
CPS (codon pair score)	Measures codons pair bias. It is defined analogously to RSCU.	It can be used to determine the level of similarity in codon pair preferences between viruses and their host.	A positive and higher score indicates preferred codon pair.	*Human*,*S. barbatus, M. musculus,G.g.domesticus, D.rerio, A.* *aegypti*, *A.gambiae*, *C.* *quinquefasciatus*, *I.scapularis* and 159 different viruses	[103 ]	[60], [61]
Frare (frequency of rare codons)	It is defined by calculating the frequency of occurrence of all codon pairs in coding sequences.	It can be used to measure codon usage and estimate the stability of exogenous genes.	A lower value of a gene indicates that that gene is an essential gene.	*E.coli*	–	[104]

The main advantages of this type of indices are that they can capture complex patterns that are not represented only by single codon distributions (such as codon pairs usage). Some of them can also consider various aspects that are currently not well understood (e.g., novel regulatory motifs and regulatory motifs related to specific regions in coding sequences) related to all aspects of gene expression. In addition, they usually do not require additional measurements other than the genome of the analyzed organism.

Their disadvantages include the fact that they are more complicated to compute than the previously mentioned indices and may require the design of efficient algorithms and more running time. In addition, some of the indices are based on or require additional prior knowledge, such as knowledge about features that affect codon bias, highly expressed genes, and more.

Therefore, it is useful to use this type of indices when there is a need to study more complex patterns in the coding region that may be related not only to translation elongation or to known intracellular phenomena.

In this type of indices, the most used indices according to our review based on the usage and number of citations, are ChimeraARS, ENcp.

## 6 Indices based on direct experimental measurements of translation and transcription elongation

**Fig. 3:**
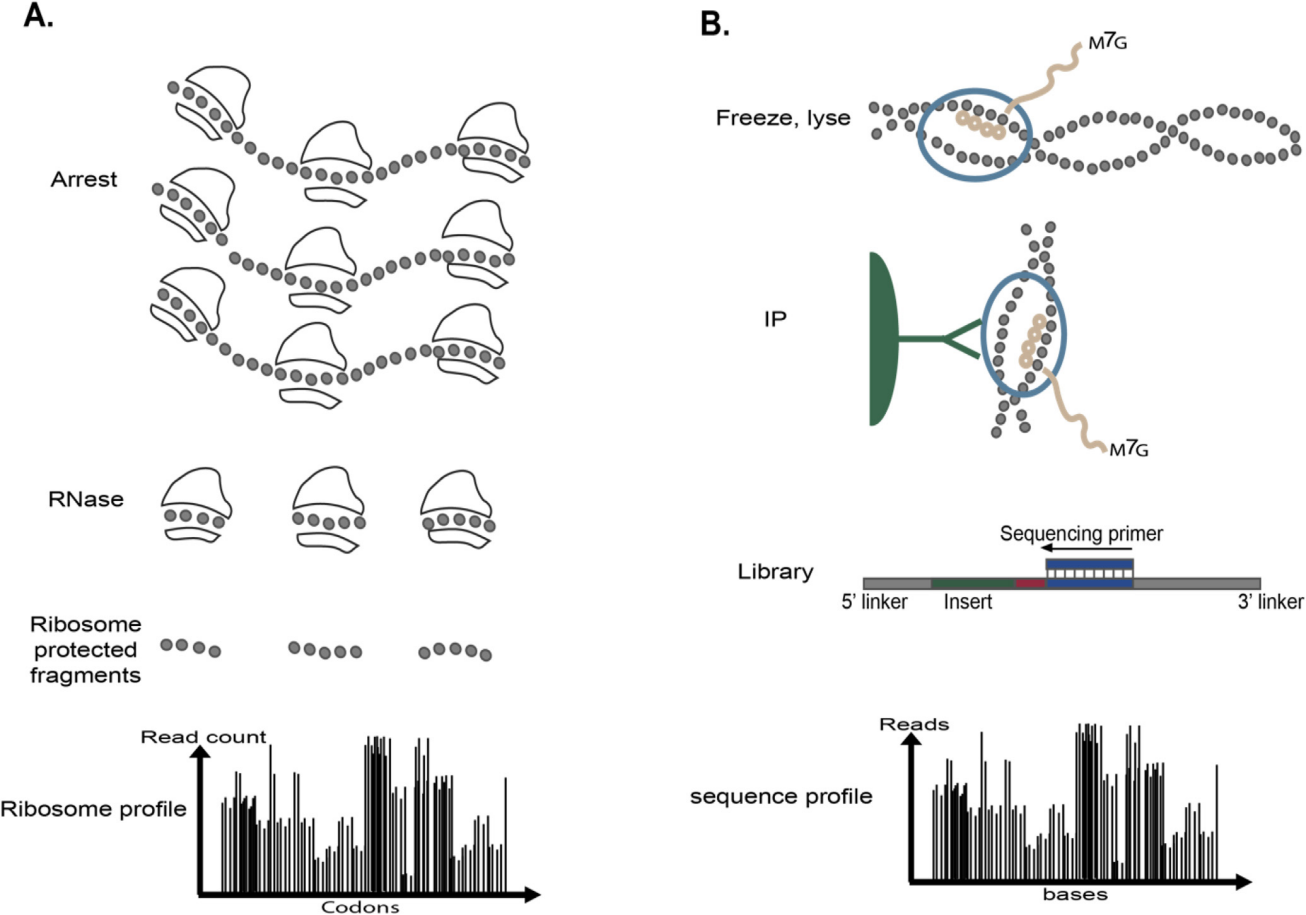
**A.** Ribosome profiling procedure. Translation of mRNAs by ribosomes is arrested, then exposed mRNA is digested. Protected mRNA footprints are then sequenced and mapped to the genome, creating for each gene its read count profile. **B.** NET-seq procedure. A culture is flash frozen and cryogenically lysed. Nascent RNA is co-purified via immunoprecipitation (IP) of the RNAPII elongation complex. Conversion of RNA into DNA results in a DNA library with the RNA as an insert between DNA sequencing linkers. The sequencing primer is positioned such that the 3′ end of the insert is sequenced. m^7^G refers to the 7-methylguanosine cap structure at the 5′ end of nascent transcripts.

Most of the CUB indices are based only on the genome of the analyzed organism. However, in many cases, the genome alone is insufficient to understand the effect of codon bias on various gene expression steps directly. In recent years, various novel experimental approaches for measuring aspects of gene expression (e.g., elongation of ribosomes and RNAP (RNA polymerase) have been developed (see Fig. 2E). One widely used experiment is the ribosome-profiling protocol; it is based on the deep sequencing of ribosomes protected mRNA fragments and enables genome-wide investigation of translation at the resolution of single codons [63] (Fig. 3A).

These types of experiments enable us to infer more direct codon bias indices related to gene expression steps such as translation elongation and transcription elongation in specific conditions.

For example, the mean typical decoding rate (MTDR) [64] is an index that aims at inferring the typical decoding rates of codons composing a certain coding region based on ribosome profiling data [63]. The index assumes that the normalized read count distribution of each codon is related to a sum of two random variables: normal (related to the typical decoding rates; where the mean of the distribution is the typical decoding rate of the codon) and exponential distributions (which is related to traffic jams and extreme biases in the experiment protocol). The parameters of these distributions are fitted to each codon separately based on the maximum likelihood approach. The MTDR of a gene is defined as the geometric mean of its codons’ typical decoding rates. Gardin et al. [65] developed a similar index of codon decoding time called 'Ribosome Residence Time' (RRT) based on a different statistical approach (average of the relative frequency distributions). Another study by Cohen et al. [66] used Native elongating transcript sequencing (Net-seq data) [67] (a procedure for capturing nascent RNA transcripts directly from live cells, Fig. 3B) to estimate the typical transcription elongation rate of the RNA polymerase related to nucleotide 5-mers, which is then used for estimating the mean transcription elongation rate of a transcript. The mean typical transcription elongation rate (MTTR) is computed per gene based on the geometric mean of the reciprocals of all its k-mer scores.

See more indices in Table 5.

**Table 5:** Codon bias indices based on direct experimental measurements of translation and transcription elongation summary.

Index type: Direct experimental measurements of translation and transcription elongation						
Index name	Specific characteristics	Uses	Values	Analyzed organism	Online tool	Reference
MTDR (Mean typical decoding rate)	Measures the codon decoding time based on ribosome profiling data.	It can be used to predict translation elongation efficiency and predict changes in translation elongation efficiency.	A higher value indicates higher translation efficiency.	*E.coli*, *s.cerevisiae* , *C.elegans*, *B.subtilis*, *M.musculus,Human*	–	[64]
RRT (Ribosome Residence Time)	Measures the codon decoding time based on ribosome profiling data.	It can be used to predict translation elongation efficiency.	Ranges from 0 to 1. A higher value indicate a slower codon decoding speed.	*S.cerevisiae*	–	[65]
MTTR (mean typical transcription elongation rate)	This index estimates the typical transcription elongation of the RNA polymerase using NET-seq data.	It can be used to estimate the average transcription elongation rate of a specific gene.	Higher values are related to selective pressure for higher elongation cycles in genes.	*S.cerevisiae*	–	

The main advantages of this type of indices include the fact that they are based on real experimental data and can directly estimate various gene expression aspects. In addition, since gene expression regulation is condition-specific, these indices are also condition-specific.

The disadvantages include the fact that they are based on complex experiments (and not on the genome alone as the indices mentioned in previous sections) that are difficult to perform and are not available for many organisms.

Therefore, it is useful to use this type of indices when the experimental data is available and when there is an interest in studying high-resolution specific gene expression steps (e.g., transcription elongation or translation elongation).

In this type of indices, the most used index according to our review based on the usage and number of citations, is MTDR**.**

## 7 A comparison between various CUB indices

In this section, we compare some of the CUB indices mentioned above over the genes of *S.cerevisiae, E.coli,* and *Human,* aiming at providing some intuition regarding the correlation between the indices and protein abundance (PA) [68].

As mentioned above, gene expression is affected by CUB and also affects its evolution [5], [6], [7]. Therefore, we expect high correlations between CUB indices and gene expression. There is a high correlation between mRNA and PA (For example, in *S.cerevisiae*, the Spearman correlation is 0.67 from our analysis). However, as codon usage is related to transcription and translation, there is usually a higher correlation between CUB indices and PA (vs. the correlation between CUB indices and mRNA). Therefore, we chose to examine the relation between CUB indices and PA.

*S.cerevisiae, E.coli,* and *Human* were the selected organisms since they are highly studied models, and there is reliable PA data available for them. In addition, this set of organisms includes both bacteria and eukaryotes in addition to both unicellular and multicellular organisms*.* For each type of indices mentioned above, at least one representative index was chosen*:* ENC [27] (non-uniform usage of synonymous codon [69]), Fop [70] (codon frequency in a reference set of genes), CAI [33] (codon frequency in a reference set of genes), CBI [36] (codon frequency in a reference set of genes), CEC [37] (codon frequency in a reference set of genes), tAI [50] (adaptation to tRNA levels and their supply [41]), nTE [51] (adaptation to tRNA levels and their supply), ChimeraARS [57] (complex patterns of codons), CPS [60], [61] (complex patterns of codons), MTDR [64] (experimental procedure). We chose indices that are possible to compute (i.e., they are clear enough to implement by ourselves or their code is available), require known and achievable data, and are computationally efficient.

We calculated the mentioned CUB indices and their Spearman correlation with PA for all *S.cerevisiae, E.coli,* and *Human* genes (for more details, see the case study section).

**Fig. 4:**
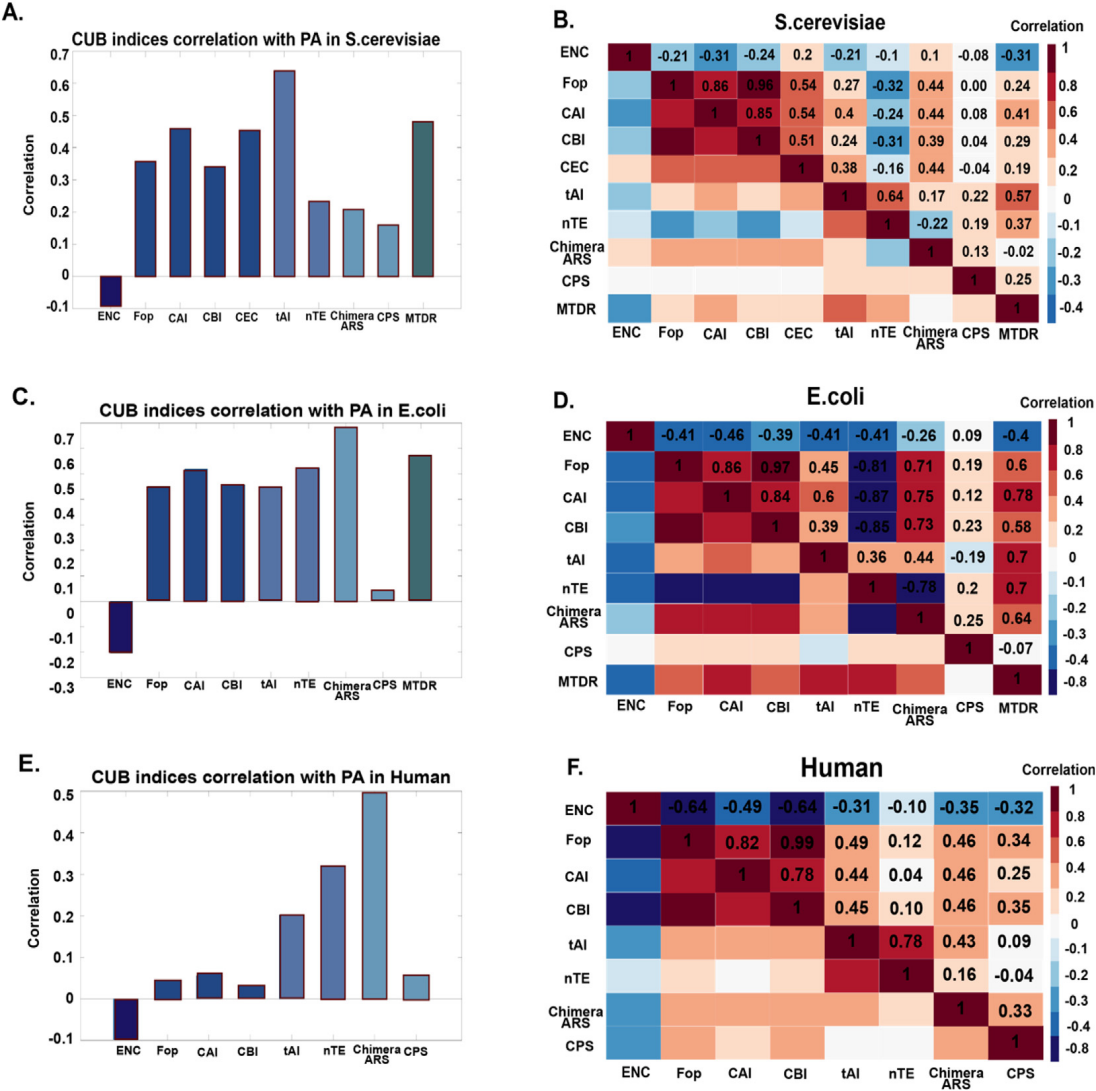
**A.** Different indices of CUB Spearman correlation with PA in *S.cerevisiae*. The indices are clustered according to types. ENC (effective number of codons), Fop (frequency of optimal codons), CAI (codon adaptation index), CBI (codon bias index), CEC (Codon-enrichment correlation), tAI (tRNA adaptation index), nTE (normalized translational efficiency), Chimera ARS, CPS (codon pair score), MTDR (mean typical decoding rate). All of the correlations between the CUB measure and PA are significant and in the right/expected direction. **B.** Spearman correlation between the different CUB indices in *S.cerevisiae***.** It can be seen that typically indices from the same type correlate better. **C.** Different indices of CUB Spearman correlation with PA in *E.coli*. The indices are clustered according to types. ENC (effective number of codons), Fop (frequency of optimal codons), CAI (codon adaptation index), CBI (codon bias index), tAI (tRNA adaptation index), nTE (normalized translational efficiency), Chimera ARS, CPS (codon pair score), MTDR (mean typical decoding rate). All of the correlations between the CUB measure and PA are significant and in the right/expected direction. **D.** Spearman correlation between the different CUB indices in *E.coli***.** It can be seen that typically indices from the same type correlate better. **E.** Different indices of CUB Spearman correlation with PA in *Human*. The indices are clustered according to types. ENC (effective number of codons), Fop (frequency of optimal codons), CAI (codon adaptation index), CBI (codon bias index), tAI (tRNA adaptation index), nTE (normalized translational efficiency), Chimera ARS, CPS (codon pair score). All of the correlations between the CUB measure and PA are significant and in the right/expected direction. **F.** Spearman correlation between the different CUB indices in *Human***.** It can be seen that typically indices from the same type correlate better.

As shown in Fig. 4A, C, and E, all of the CUB indices and PA correlations are significant and in the right/expected direction. From our analysis, indices that consider more biophysical aspects of translation (tRNA copy number, i.e., tAI), complex patterns (ChimeraARS), and uses experimental data (Ribosome profiling data, i.e., MTDR) tend to have higher correlation with PA; however, there are outliers such as the nTE. Using a reference set (highly expressed genes, according to PA) also improves the correlation with PA (CEC, CAI, FOP). Previous researches had found similar correlations and results [21], [71], [72]. It is important to mention that different indices are based on measurements (mRNA levels, tRNA copy number, and more). Therefore, these measurements and their quality can influence the index value and can by itself induce differences among the computed indices even though the indices are from the same type. Furthermore, for most of the analyzed indices, the correlations obtained for the *Human* genome were relatively lower (Fig. 4E). Possible explanations for these results include: 1) The fact that the effective population size of humans is lower than in the case of the unicellular organisms, and this results in lower evolutionary pressure and less optimal codon usage. 2) In multi-cellular organisms gene expression regulation is more complex and encoded in complex patterns in the coding region longer than single codes. Indeed, the ChimeraARS, which can capture longer patterns, performed relatively well both in the case of unicellular organisms and in the case of the *human* genome.

**Fig. 5:**
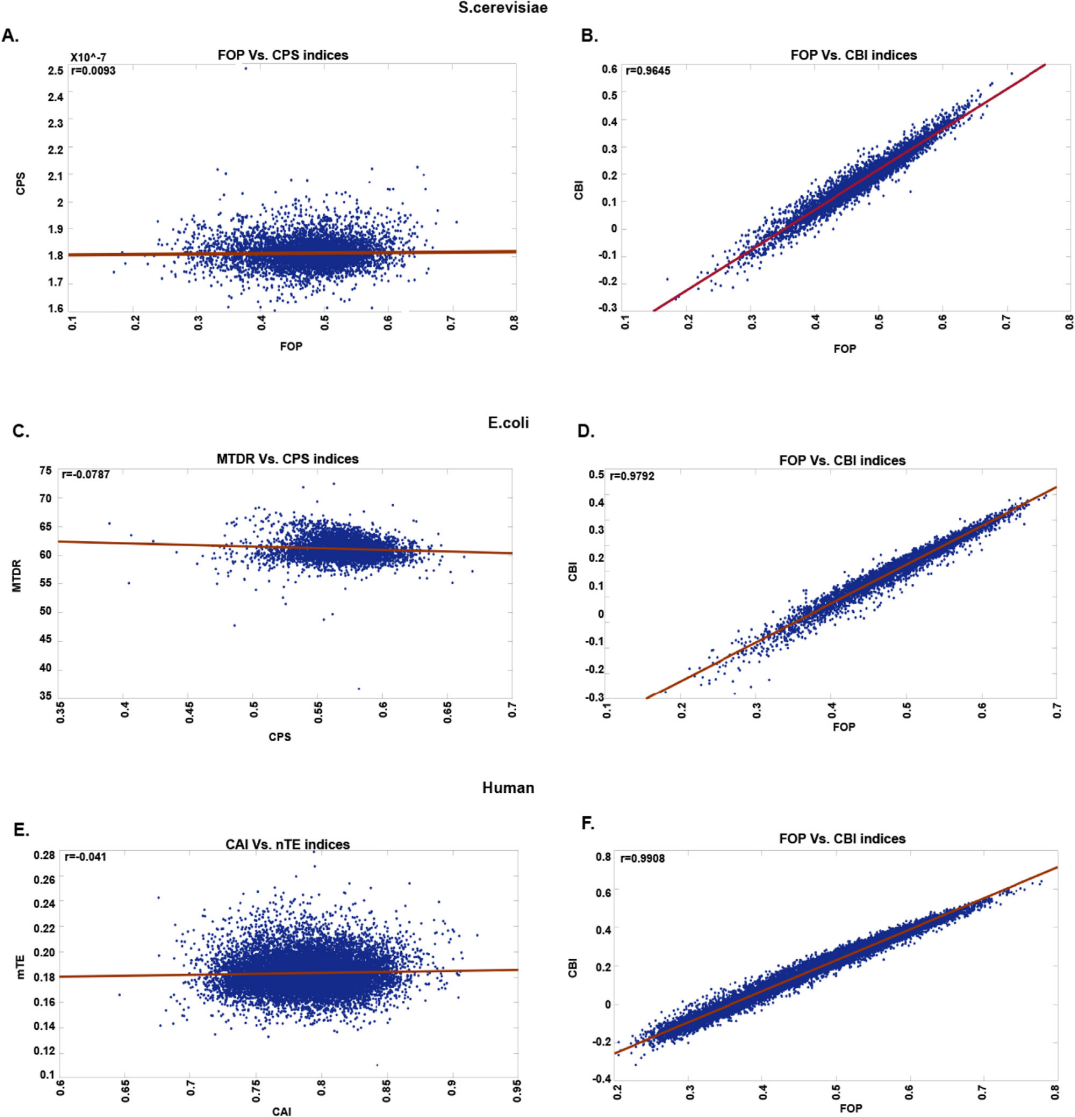
**A.** Dot plot of the lowest correlating indices FOP vs. CPS in *S.cerevisiae.***B.** Dot plot of the highest correlating indices FOP vs. CBI in *S.cerevisiae.***C.** Dot plot of the lowest correlating indices MTDR vs. CPS in *E.coli.***D.** Dot plot of the highest correlating indices FOP vs. CBI in *E.coli.***E.** Dot plot of the lowest correlating indices nTE vs. CAI in *Human.***B.** Dot plot of the highest correlating indices FOP vs. CBI in *Human.*

Afterward, we wanted to examine the relationship between the indices themselves. Therefore, we calculated the Spearman correlation between the different indices’ values for each gene (Fig. 4B, 4D, and 4F). In Fig. 5, the lowest and highest correlating indices can be seen for all analyzed organisms. When comparing the correlation between different indices, we can see from our analysis of a small number of indices that typically indices from the same type correlate better in all analyzed organisms. Moreover, most correlations are positive since the indices’ values are in the same direction and trend for most genes. A negative correlation can be seen between all indices and ENC. The negative correlation results from the index's calculation method; in all indices, a higher value indicates higher bias or higher adaptation. In the ENC index, a higher value indicates lower bias. Additional negative correlations can result from the different aspects that the different indices capture, for example, codon expected distribution, biophysical aspect, and more. Interestingly, the indices' pairs with the highest correlation were identical in all the analyzed organisms (the indices with the highest correlation are FOP and CBI). This may suggest that similar correlations between pairs of indices also appear in other organisms.

In Table 1, Table 2, Table 3, Table 4, Table 5, a short summary of many indices from all mentioned types can be seen. The reference cited is the original paper where possible. All of the indices can be used to investigate unicellular and multicellular organisms. For each index, the organism analyzed in the original paper is described, and the usage from the paper is mentioned.

## 8 Summary and discussion

We provide here a short review of CUB indices that exist today. We specifically summarized the different indices, classified them according to their type, and discussed their advantages and disadvantages. We hope that our review will help researchers in the field to choose the CUB index that fits their goals best.

Our analysis demonstrates that codon usage varies significantly among organism groups, organelles, and viruses; in addition, at least some of the indices that were examined in our review tend to correlate even though they aim at capturing different aspects. One reason for this correlation is that highly expressed genes tend to be under higher selection pressure. They tend to be more tightly regulated, and they specifically tend to have a more efficient translation, which is captured by various types of indices. Therefore, we believe that developing indices to quantify the extent of codon bias can help understand different regulatory motifs and even predict gene expression when experimental data is not available. Creating additional indices that consider known and new aspects of gene expression can improve the understanding of how evolution promotes transcripts to be more optimal to various gene expression processes in many organisms.

When using various indices, it is important to remember and consider that they may include many biases. The nucleotide composition can influence CUB indices as a result of mutational biases [27], the length of the gene (usually shorter genes tend to have higher variation due to stochastic sampling effects), the degree of codon degeneracy (affect indices that are based on reference sets), biophysical properties of the protein [105], biases that are related to the experimental data (i.e., biases in ribo-seq data [106], [107]), Secondary structure of the gene [105], and more. For example, indices that are based on the non-uniform usage of synonymous codon are strongly influenced by the length of the coding sequence (shorter genes tend to be related with higher bias), indices that are based on experimental procedures are affected by biases in the procedures themselves, and indices related to the adaptation to the tRNA pool can be biased due to the inaccurate estimation of tRNA levels which means that for different purposes, different indices are more suitable. When choosing an index, it is useful to understand the available data; for more known organisms, indices that use additional information can be used or even indices based on experimental data. For less known organisms, indices that are based solely on the genome are preferred. Moreover, there is a need to understand which aspect of gene expression is important to capture. For example, if translation elongation is studied, perhaps indices based on tRNA adaptation are preferred. Additional criteria are the level of technical expertise and computational power; we reported here both simple indices that are easier to implement and more challenging indices.

Note that as we show in our analysis and based on previous studies, there is a high correlation between some indices. In addition, many of them have been used successfully for many different objectives (e.g., gene expression prediction, molecular evolution modeling, biotechnology, biophysical modeling, etc.). Thus, it is impossible to suggests for each such objective one certain index.

Due to a large number of available indices, from our research, we can deduce that indices with embedded code or software are more likely to be used. Therefore, when creating new indices, creating a published tool can enhance the usage in the index. There are available software or online tools for different indices [31], [75], [79], [97]. For example, the CBDB (codon bias database) [91] was developed to provide a resource for researchers investigating codon bias in bacteria, facilitating comparisons between strains and species. In this database, different indices of CUB can be seen for different bacteria highly expressed genes. It is important to mention that many papers that introduce new indices have available source code or a code package for download. Still, it limits the index's usage to researches that are more computational.

From our point of view, more work needs to be done in the aspect of evaluating and compare different indices’ performance. For example, this can be done by designing sets of sequences with different levels of codon bias (based on a known statistical model) and compare the predictions of the indices to the known ground truth [108]. However, this is a non-trivial mission as the aim of the indices reported in this review in many cases is not only to estimate the non-uniform usage of codons. As mentioned, they are used, among others, to predict various aspects of gene expression, to capture specific patterns in the coding regions, and for various additional objectives. Thus, developing a good framework for such an evaluation is challenging and should consider all the different CUB indices' usages. As we suggest here, some of the indices correlate; thus, such a framework should first evaluate the level of correlation of an index to the rest of the indices.

In summary, we believe that CUB indices' main advantage is their simplicity: they provide a single number for each coding sequence. However, this is also one of their disadvantages as one number usually cannot capture the entire relevant information encoded in the coding sequence. It is important to understand the analyzed organism and the reliable and available data to choose the suitable index. Moreover, there is a need to further develop indices that are, on the one hand, simple but on the other hand can capture more information that is encoded in coding sequences, such as the fact that codon usage is not constant along the coding region.

## 9 Case study

### 9.1 Analyzed organisms

We analyzed 40 bacteria genome from the following phyla or classes: Alphaproteobacteria, Betaproteobacteria, Cyanobacteria, Deltaproteobacteria, Gammaproteobacteria, Gram-positive bacteria, Purple bacteria, Spirochaetes bacteria. In addition, we analyzed 40 chloroplast genomes, 40 eukaryotes genomes, 40 viruses from all types according to Baltimore classification genomes, and 40 mitochondria genomes. All genomes were downloaded from NCBI (https://www.ncbi.nlm.nih.gov). The complete list can be seen in Table S1.

### 9.2 Computational software

The computational analysis, including graphs creation, indices calculation, and correlation calculation, was done in MATLAB.

### 9.3 Codon frequency analysis

We examined codon frequency in different organisms, organelles, and viruses (see Fig. 1). To calculate each codon frequency, we used the following equation:

(1) $$\mathrm{Codon}\;i\;frequency=\frac{\mathrm{number}\;of\;times\;codon\;i\;apears}{\mathrm{number}\;of\;times\;the\;relevant\;amino\;acid\;apears}$$

We calculated all 64 codons frequency for all chosen organisms.

### 9.4 A comparison between various CUB indices

We compare CUB indices over *S.cerevisiae, E.coli* and, *Human* genes, aiming at providing some intuition regarding the correlation between the indices and protein abundance (PA).

First, we downloaded and extracted *S.cerevisiae*, *E.coli* and, *Human* genomes and PAs. To analyze Human PA, we averaged PA levels from 11 different tissues (brain, colon, heart, kidney, liver, lung, pancreas, plasma, saliva, skin, uterus).

Second, for each gene, we calculated the CUB index. Third, we calculated the Spearman correlation between each gene value of the chosen index and its protein abundance (Fig. 4A, 4C, and 4E).

**The chosen indices:**

We chose indices that are clear and easy enough to implement by ourselves or that their code is available, require known and achievable data, and are computationally efficient.

**Type 1:** Indices that are based on the non-uniform usage of synonymous codon:

**ENC** - Let *x_i_* be the number of synonymous codons of each type in the sequence

The number of times the AA appears in the sequence: $n=\sum_{i}^{d}x_{i}$

The frequency (/probability) of each codon is: $p_{i}=x_{i}/n$

Where:

(2) $$\hat{F}=\sum_{i}^{d}p_{i}^{2}$$

The ENC for the group of AA with degeneracy *d*:

(3) $$\hat{F_{d}}=\frac{1}{A_{d}}\sum_{i\in A_{d}}\hat{F_{i}}$$

where $A_{d}$ is the number of AA with the same degeneracy d.

The ENC for the sequence (e.g., gene):

(4) $$\mathrm{ENC}=2+\frac{9}{\hat{F_{2}}}+\frac{1}{\hat{F_{3}}}+\frac{5}{\hat{F_{4}}}+\frac{3}{\hat{F_{6}}}$$

Type 2: Indices based on codon frequency in a reference set of genes:

**FOP** – To calculate this index we used the equation:

(5) $$\mathrm{FOP}=\frac{\#optimal\;codons\;in\;sequence}{\#total\;codons\;in\;sequence}$$

Optimal codons were chosen to be the codons with maximum occurrences for each amino acid in highly expressed genes (20% of the genes with the highest PA).

**CAI** - For each codon, we calculated its weight in a reference set (20% of the genes with the highest PA):

(6) $$W_{i}=\frac{x_{i}}{max(X)}$$

where$x_{i}$ it’s the codon i frequency in the reference set and X is the frequencies of all synonymous codons of the relevant amino acid.

In the next step, for each gene j we calculated the CAI value:

(7) $${\mathrm{CAI}}_{j}=exp\left(\frac{1}{L}\sum_{l=1}^{L}ln\left(W_{l}\right)\right)$$

where L is number of codons in gene j and $W_{l}$ is the weight of codon l.

**CBI** – To compute this index we used the following equation:

(8) $$\mathrm{CBI}=\frac{N_{\mathrm{pfr}}-N_{\mathrm{rand}}}{N_{\mathrm{tot}}-N_{\mathrm{rand}}}$$

where, Npfr is the total number of occurrences of preferred codons: $N_{\mathrm{prf}}=\sum_{c\in C_{\mathrm{prf}}}N_{c}$

Cprf, is the subset of optimal codons from all codons C that are included in the analysis.

Nrand is the expected number of the preferred codons if all synonymous codons were used equally: $N_{\mathrm{rand}}=\sum_{a\in A}N_{a}\frac{{O_{a}}^{\mathrm{prf}}}{K_{a}}$.

Na is the number of occurrences of amino acid a in the sequence, ${O_{a}}^{\mathrm{prf}}$ is the number of instances of optimal codons for amino acid a, and Ka is the codon redundancy.

Ntot is the total number of amino acids in the sequence.

Preferred codons were chosen to be the codons with maximum occurrences for each amino acid in highly expressed genes (20% of the genes with the highest PA).

**CEC** – This index was used only in the analysis of *S.cerevisiae*, and the index values were taken from the original paper [37].

Type 3: Indices based on the adaptation to tRNA levels and their supply:

**tAI** – for each codon, we calculated its weight using the following equation:

(9) $$W_{i}=\sum_{j=1}\left(1-s_{\mathrm{ij}}\right)tGCN_{j}$$

Where $s_{\mathrm{ij}}$ is the affinity between codon i and anti-codon j, $\mathrm{tGC}N_{j}$ is the tRNA copy number of the tRNA with anti-codon j.

In the next step, for each gene, we calculated the tAI value:

(10) $$\mathrm{tAI}=exp(\frac{1}{L}\sum_{l=1}^{L}ln\left(W_{l}\right))$$

where L is the number of codons in the gene and $W_{l}$ is the weight of codon l.

**nTE** – In the first step, we computed tAI weights for each codon using equation (9).

In the second step, we computed the demand for each codon using the following equation:

(11) $$U_{i}=\sum_{g}n_{i,g}∙m_{g}$$

where $n_{i,g}$ is the number of times codon i appears in gene g and $m_{g}$the number of copies of gene g [47], [109].

The nTE for codon i is:

(12) $${\mathrm{nTE}}_{i}=\frac{c{\mathrm{TE}}_{i}}{{\mathrm{cU}}_{i}}$$

where $c{\mathrm{TE}}_{i}$ is the normalized tAI weight of codon i and ${\mathrm{cU}}_{i}$ is the normalized demand of codon i.

Before calculating the gene nTE, all nTE of the codons are normalized.

The nTE of a gene is:

$$\mathrm{nTE}=exp(\frac{1}{L}\sum_{l=1}^{L}ln\left({\mathrm{nTE}}_{l}\right))$$

Type 4: Indices based on 'complex' patterns of codon usage:

**ChimeraARS** – We defined highly expressed genes using 20% of the genes with the highest PA as the reference genome. To calculate this index, we used the ChimeraUGEM software [58].

**CPS** – To compute this index, first, each codon pair score is calculated using the following equation:

(14) $${\mathrm{CPS}}_{i}=ln\left(\frac{F\left(AB\right)}{\frac{F(A)∙F(B)}{F(X)∙F(Y)}∙F\left(XY\right)}\right)$$

where F(AB) is the frequency of codon pair AB, F(A) the frequency of codon A, F(B) the frequency of codon B, F(XY) is the frequency of amino acid pair XY, F(X) the frequency of amino acid X, F(Y) the frequency of amino acid Y.

The CPS score of a gene is:

$$\mathrm{CPS}=Averageallofthegene{\mathrm{CPS}}_{i}$$

Type 5: Indices based on direct experimental measurements of translation and transcription elongation:

**MTDR** – To compute this index, we used the typical decoding rate (TDR) of each codon from [110].

The MTDR of a gene is calculated using the following equation:

(16) $$\mathrm{MTDR}=exp(\frac{1}{L}\sum_{l=1}^{L}ln\left({\mathrm{TDR}}_{l}\right))$$

## Declaration of Competing Interest

The authors declare that they have no known competing financial interests or personal relationships that could have appeared to influence the work reported in this paper.
